# Incidence and Risk Factors for Severe Pneumonia in Children Hospitalized with Pneumonia in Ujjain, India

**DOI:** 10.3390/ijerph17134637

**Published:** 2020-06-27

**Authors:** Sunil Kumar Kasundriya, Mamta Dhaneria, Aditya Mathur, Ashish Pathak

**Affiliations:** 1 Department of Pediatrics, Ruxmaniben Deepchand Gardi Medical College, Ujjain 456006, Madhya Pradesh, India; praskujn8001@gmail.com (S.K.K.); mamtadhaneria@gmail.com (M.D.); dr.adityamathur121@gmail.com (A.M.); 2 All India Institute of Medical Science, Raipur 492001, Chhattisgarh, India; 3 Department of Women and Children’s Health, International Maternal and Child Health Unit, Uppsala University, SE-751 85 Uppsala, Sweden; 4 Global Health—Health Systems and Policy: Medicines, Focusing Antibiotics, Department of Public Health Sciences, Karolinska Institutet, SE-171 76 Stockholm, Sweden

**Keywords:** risk factors, community acquired pneumonia, severe pneumonia, children

## Abstract

Childhood pneumonia is a major public health problem. The aim of this prospective hospital-based study is to determine the incidence and risk factors for community-acquired severe pneumonia in children in Ujjain, India. The study includes 270 children, 161 (60%) boys and 109 (40%) girls, aged between 2 months and 5 years with World Health Organization defined and radiologically confirmed severe pneumonia. Considering the 270 children, 64% (95% confidence interval (CI) 57.9–69.4) have severe pneumonia. The following are identified as risk factors for severe pneumonia from the generalized logistic regression model: Born premature (adjusted odds ratio (AOR) 7.50; 95% CI 2.22–25.31; *p* = 0.001); history of measles (AOR 6.35; 95% CI 1.73–23.30; *p* = 0.005); incomplete vaccination (AOR 2.66; 95% CI 1.09–6.48; *p* = 0.031); acyanotic congenital heart disease (AOR 9.21; 95% CI 2.29–36.99; *p* = 0.002); home treatment tried (AOR 3.84; 95% CI 1.42–10.39; *p* = 0.008); living in a kuchha house (AOR 3.89; 95% CI 1.51–10.01; *p* = 0.027); overcrowding (AOR 4.50; 95% CI 1.75–11.51; *p* = 0.002);poor ventilation in living area (AOR 16.37; 95% CI 4.67–57.38; *p* < 0.001); and practicing open defecation (AOR 16.92; 95% CI 4.95–57.85; *p* < 0.001). Awareness of these risk factors can reduce mortality due to severe pneumonia.

## 1. Introduction

Pneumonia is the most common illness affecting infants and children globally. It is the most common cause of under-5 (U-5) mortality and contributes to 15% of U-5 mortality in 2017, killing an estimated 808,920 children [1]. World Health Organization (WHO) data indicate that acute respiratory infections (ARIs) are the second most common cause of disability-adjusted life years lost around the world [1]. During the Millennium Development Goal era from 2000 to 2015, the WHO-led ARI control program reduced the burden of pneumonia deaths from 1.7 million annual cases to 0.9 million, a 30% decrease in burden [2]. During the same time, the pneumonia mortality rate decreased by nearly 51% [3,4]. 

The ARI control program is dependent on the early identification and treatment of children with signs and symptoms suggestive of pneumonia, presuming a bacterial etiology. The WHO and United Nations Children’s Fund (UNICEF), in 2013, published the integrated Global Action Plan for Pneumonia and Diarrhea (GAPPD), which outlined a framework for ending preventable child deaths due to diarrhea and pneumonia by 2025 [2]. The GAPPD emphasizes a “protect, prevent, treat” approach and includes proven effective interventions [2]. However, estimates suggest that the sustainable development goal to eliminate preventable child deaths by 2030 will remain improbable unless deaths due to childhood pneumonia are significantly reduced [5]. 

However, more knowledge on the risk factors affecting the severity of pneumonia is required to reduce deaths from childhood pneumonia. Studies from low/middle-income countries (LMICs) have tried to identify the risk factors for severe pneumonia [6,7,8,9,10,11,12,13,14,15,16,17], but only a few studies from India have reported the risk factors for severe pneumonia [6,8,13]. More clinical studies from India are needed as the country alone contributes to 32% of the annual global burden of pediatric pneumonia [18]. India had the highest number of global U-5 deaths due to pneumonia with an estimated 508 deaths per day in 2017 [18]. No clinical study on the risk factors for child pneumonia has been published from the state of Madhya Pradesh (MP), India. Since MP has one of the highest infant and childhood mortalities in India [19], the present study has been undertaken to fill the evidence gap on the risk factors for severe child pneumonia. The aim of this study is to develop recommendations for appropriate interventions to address the demographic, socioeconomic, and environmental risk factors for severe pneumonia in children in the age range of 1–59 months in Ujjain, MP, India.

## 2. Materials and Methods 

### 2.1. Study Setting

The study was conducted from July 2015 to June 2016 in the pediatric wards of C.R. Gardi Hospital (CRGH)—a 720 bed teaching hospital attached to R.D. Gardi Medical College, Ujjain, which is managed by a charitable trust. 

### 2.2. Study Participants and Outcome Measure 

Consecutive children aged from 2 to 59 months, admitted in the pediatric ward or pediatric intensive care unit, were screened for pneumonia by two pediatricians. The pneumonia cases were defined by fast breathing or tachypnoea as delineated by the WHO (in children 2–12 months of age, breathing rate ≥50 cycles per minute; in children 12–59 months of age, breathing rate ≥40 cycles per minute) and chest indrawing [20]. 

The outcome measure for the study was “severe pneumonia” and was defined according to the WHO as a child aged 2–59 months with cough and/or difficulty breathing along with any of the danger signs such as inability to drink, persistent vomiting, convulsions, lethargy, unconsciousness, stridor in a calm child, severe acute malnutrition and hypoxemia defined as oxygen saturation (SaO2) (<90%) on fingertip pulse oximetry [21]. Apart from the above definition, other inclusion criterian were: a) First symptoms within the last 14 days and b) children with radiologically confirmed pneumonia as per WHO guidelines [17]. These children were included in the study after obtaining informed consent from the parents. Children admitted to other hospitals, those treated with intravenous or intramuscular antibiotics within the last 24 h, children with positive HIV status, and children administered steroids within the last month were excluded from the study.

### 2.3. Definitions Used in the Study

The definitions used in the study are provided in Supplementary Material Table S1.

### 2.4. Data Collection Method

SaO_2_ was measured on admission by trained clinical assistants using a fingertip pulse oximeter (ChoicemmedMD300CN356, ChoiceMMed Technology, New Delhi, India). The mother or caregiver accompanying the child fulfilling the inclusion criteria (tachypnoea plus hypoxemia on fingertip pulse oximeter) was interviewed by one of the research assistants to fill a predefined questionnaire. The questionnaire contained information on the signs and symptoms of pneumonia on admission, demographics of the child and mother, relevant history, history of the treatment received before hospitalization, and environment-related risk factors for pneumonia (Appendix A). Two independent pediatric consultants assessed each child and reached an agreement on the severity of pneumonia. All children included in the study were managed according to the India Clinical Epidemiology Network (IndiaCLEN) Task Force on Pneumonia guidelines [21]. 

### 2.5. Laboratory Investigations

Laboratory investigation, apart from X-ray for confirmation of pneumonia, included absolute leukocyte count, absolute neutrophil count, and C-Reactive Protein (CRP). Extreme leukocytosis was defined as a absolute leukocyte count above or equal to 25,000/mm and moderate leukocytosis was defined as a count between 15,000–24,999/mm [22]. Neutrophil percentage was calculated using a peripheral smear examination. The lower cut-off value for CRP was defined as 20 mg/dL [23]. Blood samples were drawn by venepuncture at time of admission. Blood cell count was performed using the 5-part coulter counter H 550 Transasia (Mumbai, India). CRP serum level was measured by the immunoturbidimetric assay using the Tulip Diagnostics Ltd. (Nagpur, India).

### 2.6. Sample Size Calculation

A study from Lucknow, India reported that nearly 20% of children presenting with fast breathing were diagnosed with severe pneumonia [24]. To calculate the sample size for the present study, we assumed the proportion of severe pneumonia as 20% or 0.2. The sample size calculation was done to detect at least 15% precision around the proportion of 20% or 0.2 with a power of 80 and a two-sided alpha of 0.05. Thus, the calculated minimum sample size was 151 children with severe pneumonia.

### 2.7. Data Management and Statistical Analysis

Data from paper forms was entered into EpiData Entry (Version 3.1, EpiData Software Association, Odense, Denmark). Data were analyzed using Stata (Version 13.0, Statacorp. TX, USA). The outcome variable was severe pneumonia. The association between independent variables (risk factors) and the outcome variable of severe pneumonia was evaluated in 2-by-2 tables using the Pearson chi-square test. The results of the 2-by-2 tables with each independent risk factor were reported as unadjusted odd ratios. 

To develop the final reported model, stepwise multivariate logistic regression models with backward elimination of the independent variables having *p* values more than 0.1 were used. The independent variables of age and gender were kept in the final model even if their *p* values were more than 0.1. The final model reports the adjusted odd ratios (AORs) and their 95% confidence intervals (CIs). A *p* value of less than 0.05 was considered significant. Model discrimination was conducted using the C-statistics-receiver-operating-characteristics curve [25]. The predictive power of the final and initial models were compared using the following two statistical methods: Akaike information criterion (AIC) calculated as [AIC = −2 log maximum likelihood + 2× degrees of freedom (df)] [26]; Bayesian information criterion (BIC) which was calculated as [BIC = −2 log maximum likelihood + log (sample size) × df)] [27]. The study was approved by the institutional ethics committee of R.D. Gardi Medical College, Ujjain (Institution Ethics Committee reference number 2013).

## 3. Results

**Figure 1:**
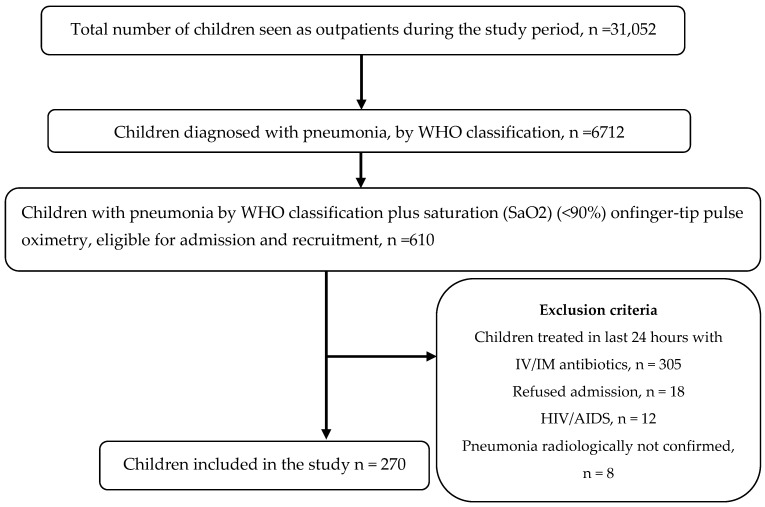
Flow chart of the patient recruitment process. WHO- World Health Organization, IV-Intravenous, IM-Intramuscular, HIV-Human Immunodeficiency Virus, AIDS-Acquired Immunodeficiency Syndrome.

During the study period, 270 children with pneumonia (60% boys and 40% girls), with the mean [±standard deviation (SD)] age of 18.5 (± 17.5) months were enrolled. Figure 1 provides the flow chart of the patient recruitment process. Considering the 270 children, 172 children were diagnosed with severe pneumonia, and the remaining 98 children had pneumonia according to the WHO classification. The prevalence of severe pneumonia was, thus, 63.7% (95% CI 57.9–69.4). The signs and symptoms at the time of admission are shown in Table 1.

The mean (±SD) duration of signs and symptoms before admission was 4 (±2.1) days. A total of 38 (14%) children required ventilatory support for a mean (±SD) duration of 36 (±5) h. Complicated pneumonia was diagnosed in 17 (6%) children, and 16 (6%) children died during the hospital stay.

### 3.1. Results of Bivariate Analysis

The association of socio-demographic and birth-related risk factors with severe pneumonia is depicted in Table 2. The table depicts that age less thanone-year, rural residence, premature birth (before 37 weeks of gestational age), and non-working mother increased the risk of severe pneumonia. 

The association of history-related risk factors with severe pneumonia is shown in Table 3. Factors such as non-exclusive breast feeding in the first 6 months, lack of vitamin A supplementation, incomplete immunization, kerosene ingestion in the past 1 month, hospitalization in the past 1 month, and trial of home remedy for present episode of pneumonia increased the risk of severe pneumonia (Table 3). 

The association between environmental covariates and severe pneumonia is depicted in Table 4. Factors such as nuclear family, kuccha house, overcrowding, indoor air pollution, poor ventilation in living area, household tuberculosis contact, and open-air defecation increased the risk of severe pneumonia.

### 3.2. Results of Total Leukocyte Count, Neutrophil Percentage and CRP

The mean (±standard deviation) TLC count among children with pneumonia and severe pneumonia was 13,425(± 389); 95% CI 12,653–14,197 and 13,851 (±333); 95% CI 13,194–14,508, respectively (*p* <0.001). Considering 270 children, 52% (*n* = 112) had normal leukocyte counts, 41% (*n* = 112) had moderate leukocytosis, and 7% (*n* = 18) had extreme leukocytosis. Severe pneumonia patients had significantly more moderate leukocytosis compared to pneumonia patients (78% versus 22%, respectively, *p* <0.05). Extreme leukocytosis was observed more commonly in pneumonia (61%) versus severe pneumonia (39%), but the difference was not statistically significant (*p* = 0.48). The mean neutrophil count among children with pneumonia and severe pneumonia was 68 (±1.64); 95% CI 66–72 and 68 (±1.27); 95% CI 66–71. The difference was not statistically significant (*p* = 0.051). 

The mean CRP value among children with pneumonia and severe pneumonia was 14.13 (±0.59); 95% CI 12.95–15.32 and 21.38 (±0.56); 95% CI 20.26–22.51, respectively (*p* < 0.001). The cut-off of 20 mg/L had a sensitivity of 63%, specificity of 90%, positive predictive value of 92%, and negative predictive value of 58% and correctly classified non severe pneumonia from severe pneumonia in 73% of cases. 

### 3.3. Results of Multivariate Logistic Regression Analysis

Table 5 depicts the results of the backward logistic regression model. The risk factors identified in the final model included: age (continuous variable); gender (boys versus girls); born premature (no versus yes); history of measles in the last 6 months (no versus yes); incomplete vaccination (no versus yes); acyanotic congenital heart disease (absent versus present); home treatment tried (yes versus no);type of home (pukka versus kuccha); overcrowding (no versus yes); poor ventilation in living area (no versus yes); open defecation (no versus yes).

### 3.4. Model Discrimination

The initial model characteristics were df = 29, AIC =183.6, and BIC = 287.9, whereas the final model characteristics were df = 12, AIC = 171.6, BIC = 214.8. Therefore, the predictive power of the final model was significantly better than that of the initial model.

## 4. Discussion

The present study included children with WHO-defined symptoms and other signs to define pneumonia and severe pneumonia [2]. The other included signs which were part of the initial assessment, and whose values were ascertained by other studies, were cyanosis, toxic look, severe pallor (Hb<7 gm/dL), severe dehydration, shock, and meningeal irritation [28,29,30]. 

During our study, severe pneumonia was more common in children between two and 12 months of age compared with children between 13 and 60 months of age. The global prevalence of pneumonia is highest in the age group of 1–4 years [18]. Children residing in rural areas were more affected with severe pneumonia compared with children living in urban areas, and the condition showed a marked male predilection. Similar findings have been reported in other regions of India and other neighboring countries like Bhutan and Nepal [31,32]. 

The present study reported that prematurely born children had an increased risk of severe pneumonia. Similar results were reported in another community-based study [33]. The underlying reason could be the effects of premature birth, such as poor infant feeding, and the resultant failure of childhood growth. A decrease in growth failure indices such as stunting and wasting can bring about a significant decrease in pneumonia mortality [18]. Pneumonia developed in 4–9.6% of children with post-measles infection, depending on whether they received antibiotic treatment [34]. This cause of pneumonia, thus, can be prevented by prioritization of measles vaccination along with pneumococcal conjugate vaccine (PCV) and haemophilus influenzae type B (HIB) vaccine. PCV was not introduced in the duration of the study; however, the HIB vaccine was given as part of the pentavalent vaccine. During the present study, the children that did not receive vaccines covered in the expanded program of immunization had an increased risk of severe pneumonia. Respiratory infections were less common among children who were completely immunized than in children with incomplete immunization [18]. The increased global HIB vaccination between 1990 and 2017 reduced the U-5 mortality by 11.4%, which is the maximum among all known interventions [18]. Children having congenital heart disease (CHD) had an increased risk of severe pneumonia in our study. A left-to-right shunt is expected to have an increased risk of pneumonia and heart failure. A Nigerian study, which included 121 children with pneumonia, reported 14 (11.5%) having a CHD [35]. Children who received any home treatment had an increased risk of severe pneumonia in the present study. This could be due to greater prescription of antibiotics in the study area [36,37]. Treatment with inappropriate antibiotics could have increased the risk of severe pneumonia. 

Among the environmental risk factors, children living in kuccha houses had an increased risk of severe pneumonia. Kuccha houses are typically built by the poor and is a recognized risk factor for pneumonia in other Asian countries as well [31,32]. Overcrowding and poorly ventilated living areas were identified as risk factors for severe pneumonia. Higher levels of outdoor pollution in urban areas [18,38], high levels of solid fuel use causing indoor pollution in rural areas, and poorly constructed houses were the main reasons for the increased risk of pneumonia in children in our study [18,39]. LMICs that implemented measures to decrease exposure to household air pollution reduced pneumonia mortality by around 8%, but increased exposure to ambient air pollution increased mortality by around 4% [18]. Although open air defecation is not directly related to childhood pneumonia, it may be a common risk factor for both diarrhea and pneumonia as proposed by the WHO [2].

The present study evaluated host biomarkers like theTLC count, which was useful only in the presence of moderate leukocytosis. Generally, like the conclusion in other studies, it can be concluded that TLC and neutrophil percentage were not useful in distinguishing pneumonia from severe disease [40]. However, a CRP cut-off of 20 mg/L had a high specificity (90%) and positive predictive value (92%) to distinguish non-severe from severe disease. Thus, a CRP more than 20 mg/L might have some utility in predicting severe pneumonia in our and similar settings.

A primary limitation of this study was that the study did not ascertain the etiology of pneumonia in children, because it was not the primary study objective. Other limitations included the absence of blood investigations of the children due to the non-specific nature of the leukocyte count and C-reactive proteins in distinguishing pneumonia from severe pneumonia and the non-presentation of antibiotic use data as all children were managed using standard IndiaCLEN treatment guidelines. Another limitation of the study is the data are from a single teaching hospital. The results are generalizable to similar resource-limited settings, but hospital-to-hospital variations are expected.

## 5. Conclusions

Childhood pneumonia has been identified as the major “forgotten killer of children” by UNICEF and the WHO. Multiple modifiable risk factors for severe pneumonia have been identified in this study. Pediatricians and other health care workers, including the grassroot health workers, should be aware of these risk factors of severe pneumonia when managing the patient with pneumonia. The management of the modifiable risk factors may reduce mortality due to severe pneumonia.

## Figures and Tables

**Table 1:** Signs and symptoms at presentation in hospitalized children with severe pneumonia (*n* = 270) in Ujjain, India.

Signs/Symptoms	Total *n* = 270 (%)	Pneumonia *n* = 98 (%)	Severe Pneumonia *n* = 172 (%)
Fast breathing ^a^	258 (95)	96 (98)	172 (100)
Chest indrawing/difficulty in breathing ^a^	255 (94)	93 (95)	162 (94)
Not able to drink ^a^	238 (88)	85 (87)	153 (89)
Persistent vomiting ^a^	159 (59)	42 (43)	117 (68)
Convulsions ^a^	11 (4)	5 (6)	6 (3)
Lethargic/Unconscious ^a^	39 (14)	19 (19)	20 (12)
Stridor in a calm child ^a^	28 (10)	8 (8)	20 (12)
Severe acute malnutrition ^a^	113 (42)	77 (79)	86 (21)
Cyanosis ^b^	42 (16)	2 (2)	40 (23)
Toxic look ^b^	43 (16)	22 (22)	21 (12)
Severe pallor (Hb<7 gm/dL) ^b^	41 (15)	17 (17)	24 (14)
Severe dehydration ^b^	34 (13)	3 (3)	31 (18)
Shock ^b^	21 (8)	2 (2)	19 (11)
Meningeal irritation ^b^	17 (6)	12 (12)	5 (3)
Crepitations	199 (74)	57 (58)	142 (83)
Low grade fever (37 °C–38 °C)	188 (70)	55 (56)	133 (77)
High grade fever (>38 °C)	82 (30)	43 (43)	39 (23)
Diarrhea	91 (34)	16 (16)	75 (44)
Significant lymphadenopathy	84 (34)	44 (16)	40 (23)
Running nose	68 (25)	14 (14)	54 (31)
Wheeze	54 (20)	27 (28)	27 (16)
Ear discharge	39 (14)	19 (19)	20 (12)

^a^ danger signs according to WHO; ^b^ other danger signs, %column percentage.

**Table 2:** Bivariate associations of socio-demographic and birth-related covariates with severe pneumonia in hospitalized children (*n* = 270) in Ujjain, India.

Independent Variable	Total *n* = 270 (%) ^#^	Severe Pneumonia *n* = 172(%) ^#^	Pneumonia *n* = 98, (%) ^#^	OR	95% CI	*p* Value
**Age (in months)**						
2–12	149 (55)	83 (48)	66 (67)	R	R	-
13–60	121 (45)	89 (52)	32 (33)	2.21	1.31–3.71	0.003
**Gender**						
Boys	161 (60)	103 (60)	58 (59)	R	R	-
Girls	109 (40)	69 (40)	40 (41)	0.97	0.58–1.60	0.910
**Residence**						
Urban	100 (37)	56 (33)	44 (45)	R	R	-
Rural	170 (63)	116 (67)	54 (55)	1.68	1.01–2.81	0.044
**Born premature**						
No	56 (21)	9 (5)	47 (48)	R	R	-
Yes	214 (79)	163 (95)	51 (52)	16.6	7.65–36.38	<0.001
**Born term low birth weight**						
No	214 (79)	147 (85)	67 (68)	R	R	-
Yes	56 (21)	25 (15)	31 (32)	0.36	0.20–0.67	0.001
**Mother’s education**						
Illiterate	26 (10)	11 (6)	15 (15)	R	R	-
Upto secondary	225 (83)	148 (86)	77 (79)	2.62	1.14–5.98	0.022
Graduate	19 (07)	13 (8)	06 (6)	2.95	0.85–10.22	0.087
**Mother’s occupation**						
Working	33(12)	9(5)	24 (24)	R	R	-
Nonworking	237(88)	163(95)	74 (76)	0.17	0.07–0.38	<0.001

OR–odds ratios, CI– Confidence interval, ^#^–Column percentage, R– referent category.

**Table 3:** Bivariate associations between past history-related covariates and severe pneumonia in hospitalized children (*n* = 270) in Ujjain, India.

Independent variable	Total *n* = 270 (%) ^#^	Severe Pneumonia *n* = 172 (%) ^#^	Pneumonia *n* = 98, (%) ^#^	OR	95% CI	*p* Value
**Exclusive breastfeeding**						
Yes	216 (80)	125 (73)	91 (93)	R	R	-
No	54 (20)	47 (27)	7 (7)	4.88	2.11–11.30	<0.001
**Vitamin A supplementation**						
Given	225 (83)	139 (81)	86 (88)	R	R	-
Not given	45 (17)	33 (19)	12 (12)	1.70	0.83–3.47	0.144
Measles *						
No	209 (77)	123 (72)	86 (88)	R	R	-
Yes	61 (23)	49 (28)	12 (12)	2.85	1.43–5.68	0.003
**Incomplete vaccination**						
No	132 (49)	59 (34)	73 (74)	R	R	-
Yes	138 (51)	113 (66)	25 (26)	5.59	3.21–9.71	<0.001
Kerosene ingestion **						
No	235 (87)	143 (83)	92 (94)	R	R	-
Yes	35 (13)	29 (17)	6 (6)	3.10	1.24–7.78	0.015
Antibiotic treatment **						
No	204 (76)	125 (73)	79 (81)	R	R	-
Yes	66 (24)	47 (27)	19 (19)	1.56	0.85–2.85	0.146
Hospitalization **						
No	212 (79)	125 (73)	87 (89)	R	R	-
Yes	58 (21)	47 (27)	11 (11)	2.97	1.46–6.05	0.003
**Acyanotic congenital heart disease**						
Absent	241 (89)	150 (87)	91 (93)	R	R	-
Present	29 (11)	22 (13)	7 (7)	1.90	0.78–4.64	0.153
**Home treatment tried**						
Yes	86 (32)	26 (15)	60 (61)	R	R	-
No	184 (68)	146 (85)	38 (39)	8.86	4.95–15.87	<0.001

OR—odds ratios, CI—Confidence interval, * history of measles in the last 6 months, ** Measles history during the past month, ^#^— Column percentage, R—referent category.

**Table 4:** Bivariate associations between environmental covariates and severe pneumonia in hospitalized children (*n* = 270) in Ujjain, India.

Independent Variable	Total *n* = 270 (%) ^#^	Severe Pneumonia *n* = 172 (%)	Pneumonia *n* = 98, (%)	OR	95%CI	*p* Value
**Type of family**						
Nuclear	136(50)	68(40)	68(69)	R	R	-
Joint	134(50)	104(60)	30(30)	3.46	2.04–5.87	<0.001
**Number of family members**						
Upto 5	131 (49)	68 (40)	63 (64)	R	R	-
5–10	116 (43)	86 (50)	30 (31)	2.65	1.51–4.55	<0.001
>10	23 (8)	18 (10)	5 (5)	3.33	1.61–9.51	0.024
**Type of home**						
Pukka	101(37)	56(33)	45(46)	R	R	-
Kuccha	169(63)	116(67)	53(54)	1.75	1.05–2.92	0.030
**Overcrowding**						
No	82(30)	32(19)	50(51)	R	R	-
Yes	188(70)	140(81)	48(49)	4.55	2.62–7.91	<0.001
**Indoor air pollution**						
No	73(27)	30(17)	43(44)	R	R	-
Yes	197(73)	142(83)	55(56)	3.70	2.11–6.48	<0.001
**Poor ventilation in living area**						
No	69 (26)	14 (8)	55 (56)	R	R	-
Yes	201 (74)	158 (92)	43 (44)	14.43	7.33–28.39	<0.001
**Contact with TB**						
No	44(16)	20(12)	24(24)	R	R	-
Yes	226(84)	152(88)	74(76)	2.46	1.28–4.74	0.007
**Smoking at home**						
No	112(41)	72(42)	40(41)	R	R	-
Yes	158(59)	100(58)	58(59)	0.95	0.57–1.58	0.867
**Drinking water source**						
Underground water	112 (41)	69 (40)	43 (44)	R	R	-
Tap water	158 (59)	103 (60)	55 (56)	1.16	0.70–1.92	0.547
**Open defecation**						
No	176 (65)	90 (52)	86 (88)	R	R	-
Yes	94 (35)	82 (48)	12 (12)	6.52	3.32–12.81	<0.001

OR—odds ratios, CI—Confidence interval, ^#^—Column percentage, R—referent category.

**Table 5:** Multivariate analyses of socio-demographic, past treatment-related, environmental, and sign- and symptom-related risk factors of severe pneumonia in hospitalized children (*n* = 270) in Ujjain, India.

Independent Variables	AOR	95% CI	*p* Value
Age in months ^#^(continuous)	1.00	0.97–1.02	0.832
Gender ^#^(boys versus girls)	0.74	0.30–1.79	0.514
Born premature (no versus yes)	7.50	2.22–25.31	0.001
Measles *(no versus yes)	6.35	1.73–23.30	0.005
Incomplete vaccination(no versus yes)	2.66	1.09–6.48	0.031
Acyanotic congenital heart disease(no versus yes)	9.21	2.29–36.99	0.002
Home treatment tried (yes versus no)	3.84	1.42–10.39	0.008
Type of home(pukka versus kuccha)	3.89	1.51–10.01	0.027
Overcrowding (no versus yes)	4.50	1.75–11.51	0.002
Poor ventilation in living area(no versus yes)	16.37	4.67–57.38	<0.001
Open defecation(no versus yes)	16.92	4.95–57.85	<0.001

AOR– adjusted odds ratios, ^#^ Adjusted for age and sex, * History of measles in the last 6 months.

## Supplementary Materials

The following are available online at https://www.mdpi.com/1660-4601/17/13/4637/s1, Table S1: title-Definitions used in the study.

## Appendix A

**(A). Basic Demographic Information**

1. Enrolment number ______________________
2. Date of the form filling _____________________
3. Name ______________ Surname ___________
4. Date of birth ____________________
5. Age in months ________________
6. Sex       Male       Female
7. IPD number
8. Date of admission
9. Date of discharge/death
10. Residence       Rural        Urban

**(B). Signs and symptoms at presentation in hospitalized children with severe pneumonia**

Signs/symptoms	**Yes**	**No**
Fast breathing		
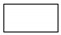
Chest indrawing/difficulty in breathing		
Not able to drink		
Persistent vomiting		
Convulsions		
Lethargic/Unconscious		
Stridor in a calm child		
Severe acute malnutrition		
Cyanosis		
Toxic look		
Severe pallor (Hb<7 gm/dL)		
Severe dehydration		
Shock		
Meningeal irritation		
Crepitations		
Fever [Low grade fever (37 °C–38 °C) High grade fever (>38 °C)]		
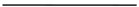
Diarrhea		
Significant lymphadenopathy		
Running nose		
Wheeze		
Ear discharge		

**(C). Socio-demographic and birth related co-variates**

	**Check Box**
**Born premature**	
No	
Yes	
**Born term low birth weight**	
No	
Yes	
**Mother’s education**	
Illiterate	
Up-to secondary	
Graduate	
**Mother’s occupation**	
Working	
Nonworking	

**(D). Past history related co-variates with severe pneumonia**

	**Check Box**
**Exclusive breastfeeding (till 6 months of age)**	
Yes	
No	
**Vitamin A** **supplementation**	
Given	
Not given	
**Measles**	
No	
Yes	
**Incomplete vaccination**	
No	
Yes	
**Kerosene ingestion**	
No	
Yes	
**Antibiotic treatment**	
No	
Yes	
**Hospitalization**	
No	
Yes	
**Acyanotic congenital heart disease**	
Absent	
Present	
**Home treatment tried (other than self-medication)**	
Yes	
No	

**(E). Environmental covariates**

**Type of family**	**Check Box**
Nuclear	
Joint	
**Number of family members**	
Upto 5	
5–10	
>10	
**Type of home**	
Pukka	
Kuccha	
**Overcrowding**	
No	
Yes	
**Indoor air pollution**	
No	
Yes	
**Poor ventilation in living area**	
No	
Yes	
**Contact with TB**	
No	
Yes	
**Smoking at home**	
No	
Yes	
**Drinking water source**	
Underground water	
Tap water	
**Open defecation**	
No	
Yes	

**(F). Investigation:**

Hb	__________
TLC	__________
DLC	__________
I/T ratio	__________
Platelet count	__________
CRP	__________

**(G). Anthropometry**

**S.No.**	**Parameters**	**Actual**	**Expected**
1.	Weight (in Kgs)		
2.	Height		
3.	Head Circumference		
4.	Mid arm Circumference		
