# Supplementary material for: Incidence and Risk Factors for Severe Pneumonia in Children Hospitalized with Pneumonia in Ujjain, India
Source: Int J Environ Res Public Health. 2020 Jun 27;17(13):4637. doi: 10.3390/ijerph17134637 (PMC7369688; doi:10.3390/ijerph17134637)
Supplement: Supplementary file 1 [file ijerph-17-04637-s001.pdf]

Table S1. Definitions used in the study.

Term	Definition	Reference
Nuclear Family	A family that consisted of a married couple and their children occupying the same dwelling space	[22]
Joint Family	A joint family consisted of more than one married couple and their children who lived together in the same household and shared a common kitchen	[22]
Overcrowding	Overcrowding was considered to be present if two persons, above 9 years of age, of opposite sexes, not husband and wife, had to sleep in the same room	[22]
A Pucca House	A pucca house is one, which has walls and roof made of burnt bricks/stones packed with lime or cement	[23]
A Kutcha House	A kutcha house was one with the walls and/or roof made of material such as un-burnt bricks, bamboos, mud, grass, reeds, thatch, loosely packed stones, etc.	[23]
Exclusive Breast-Feeding	Breast-feeding was considered exclusive if the infant has received only breast milk from his/her mother or a wet nurse, or expressed breast milk, and no other liquids or solids	[24]
Severe Acute Malnutrition (SAM)	SAM was assessed in children between the ages of 6 month to 5 years according to Indian Academy of Pediatrics consensus statement for diagnosis and management of SAM	[25]
Complicated Pneumonia	Was defined as an infection involving the lung parenchyma, which was complicated by one or more of the following: parapneumonic effusion, empyema, necrotizing pneumonia, abscess, pneumothorax, and bronchopleural fistula	[26]
